# Aetiology and 30-Year Long-Term Outcome of Children with Cardiomyopathy Necessitating Heart Transplantation

**DOI:** 10.3390/jpm10040251

**Published:** 2020-11-27

**Authors:** Martin Zschirnt, Josef Thul, Hakan Akintürk, Klaus Valeske, Dietmar Schranz, Susanne Skrzypek, Matthias Müller, Christian Jux, Andreas Hahn, Stefan Rupp

**Affiliations:** 1 Pediatric Heart Center, Department of Pediatric Cardiology and Congenital Heart Disease, University Hospital Giessen, Justus Liebig Universität Giessen, 35390 Giessen, Germany; martin.zschirnt@paediat.med.uni-giessen.de (M.Z.); josef.thul@paediat.med.uni-giessen.de (J.T.); dietmar.schranz@paediat.med.uni-giessen.de (D.S.); susanne.skrzypek@paediat.med.uni-giessen.de (S.S.); Matthias.mueller@chirurgie.uni-giessen.de (M.M.); Christian.Jux@paediat.med.uni-giessen.de (C.J.); 2 Pediatric Heart Center, Department of Pediatric Cardiac Surgery, University Hospital Giessen, Justus Liebig Universität Giessen, 35390 Giessen, Germany; Hakan.Akintuerk@chirugie.uni-giessen.de (H.A.); Klaus.Valeske@chirurgie.uni-giessen.de (K.V.); 3 Department of Child Neurology, Justus-Liebig University, 35390 Giessen, Germany; Andreas.Hahn@paediat.med.uni-giessen.de

**Keywords:** pediatric heart transplantation, cardiomyopathies, Long-Term-Outcome, personalized medicine

## Abstract

Studies assessing the long-term outcome after heart transplantation HTX in patients with cardiomyopathy (CM) in the paediatric age range are rare. The aim of this study was to determine the survival rate of children with CM undergoing HTX and to analyse how aetiology of cardiomyopathy influenced morbidity and mortality. We retrospectively analysed the medical records of children; who were transplanted in our centre between June 1988 and October 2019. 236 heart transplantations were performed since 1988 (9 re-transplants). 98 of 227 patients (43.2%) were transplanted because of CM. Survival rates were 93% after 1; 84% after 10 and 75% after 30 years. Overall; the aetiology of CM could be clearly identified in 37 subjects (37.7%). This rate increased up to 66.6% (12/19) by applying a comprehensive diagnostic workup since 2016. The survival rate was lower (*p* < 0.05) and neurocognitive deficits were more frequent (*p* = 0.001) in subjects with systemic diseases than in individuals with cardiac-specific conditions. These data indicate that the long-term survival rate of children with CM after HTX in experienced centers is high. A comprehensive diagnostic workup allows unraveling the basic defect in the majority of patients with CM undergoing HTX. Aetiology of CM affects morbidity and mortality in subjects necessitating HTX.

## 1. Introduction

Heart transplantation (HTX) is the final therapeutic option for children with terminal heart failure. Besides severe congenital heart defects (CHD), cardiomyopathy (CM) represents an increasing indication for HTX. CM is defined as a cardiac dysfunction based on electrical or mechanical malfunction in the absence of CHD or abnormal loading condition [1]. The incidence of CM in the pediatric age range is 1.1–1.5 per 100,000 [2,3]. According to cardiac morphology, different forms such as dilative CM (DCM) (incidence: 0.57 per 100,000 children), hypertrophic CM (HCM) (incidence 0.47 per 100,000 children), non-compaction CM (NCM), restrictive CM (RCM), arrhythmogenic right ventricular CM (ARVC), and non-classifiable CM [2,3,4,5,6] can be distinguished. The aetiology of CM is heterogeneous. In children, viral infections (myocarditis), defects of cardiac-specific proteins (e.g., MYH7, MYBPC3), systemic metabolic or neurodegenerative disorders (e.g., Pompe disease, Friedreich ataxia) and systemic genetic conditions (e.g., RASopathies) are common causes [7,8,9].

The International Society for Heart and Lung Transplantation (ISHLT) reported a 15-year survival rate for children after HTX of 50 to 75% [10,11,12,13], and of 37% after 25 years [14]. Just a few studies have discriminated between patients with CHD and CM [13,15,16]. The 19th ISHLT report stated an overall survival rate of 88% for children with CM compared to 79% for patients with CHD by 3 years post-transplant, and of 70% and 68% 10 years after HTX [16]. Rarely reports distinguished between specific types of CM such as HCM, DCM, and RCM [17], and only one study analysed the results of HTX in children with CM due to a specific aetiology [18]. Until now, no study has systematically analysed long-term survival of children in relation to the cause of CM.

Improved understanding of the pathomechanisms underlying CM and advances in genetic testing has facilitated unraveling the etiology of CM. In a recent study for example, the exact basic genetic defect could be clarified in about 80% of children with HCM by a sophisticated multidisciplinary diagnostic approach [19]. 

Identifying the exact aetiology of CM in children awaiting a new heart can be important since the primary disease resulting in cardiac failure could also affect long term outcome of transplantation. While the underlying condition will probably not negatively influence long-term prognosis in subjects with defects of sarcomeric proteins; progressive diseases such as mitochondrial disorders and muscular dystrophies, or systemic disorders like RASopathies could predispose to complications or increase the risk of early organ loss and neurocognitive deficits.

The aims of this study were to assess the long-term survival of heart transplanted children with CM, and to investigate whether the aetiology causing terminal cardiac failure affects the prognosis. To accomplish this, we retrospectively analysed a larger cohort of children undergoing HTX in our centre within the last 30 years.

## 2. Patients and Methods 

We reviewed the database of the Pediatric Heart Center Giessen for patients who underwent HTX between 1988 and 2019. This retrieved 236 children including 9 who have been re-transplanted. 98 of 227 patients (43.2%) were transplanted because of CM. The following items were extracted from the medical records: type of CM (DCM, HCM, RCM, non-classifiable CM, ARVC), age at transplantation, age at death, cause of CM, all information about diagnostics performed to elucidate the basic defect of CM and potential co-morbidities such as cognitive or neurological deficits.

Based on this information the cause of CM was classified as cardiac-specific, systemic, other, or unknown. Neurocognitive dysfunction is regarded as an important co-morbidity in children with CHD [20], that principally can increase the risk of organ loss [21]. In this study, we used this parameter as a marker for morbidity. A cognitive deficit was assumed when the patient attended a school for mentally handicapped persons and/or in case a neuropsychological testing had revealed an intelligence quotient below the normal range (<85). A neurological deficit was diagnosed if a neurological symptom significantly limited the patient’s ability to perform daily life activities (e.g., blindness, ataxia, hemiparesis).

**Figure 1:**
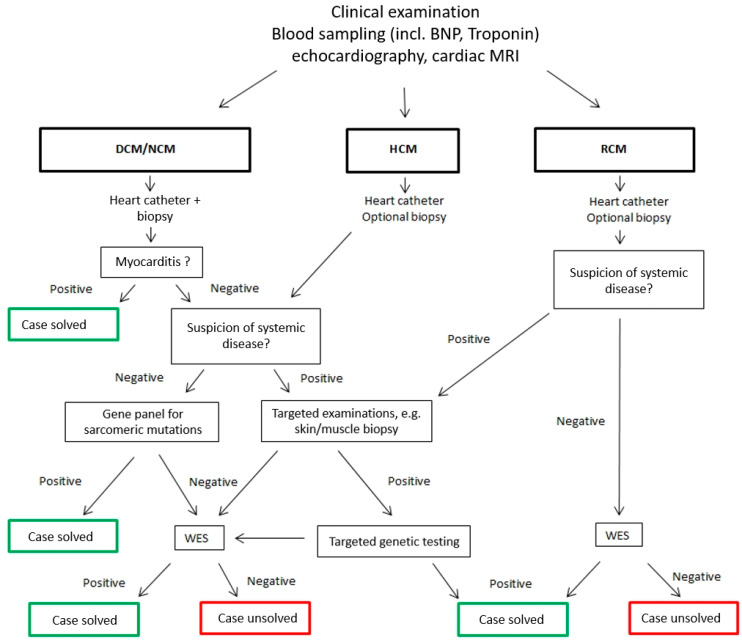
Diagnostic algorithm for subjects with cardiomyopathy. DCM = Dilated cardiomyopathy, HCM = Hypertrophic cardiomyopathy, RCM = Restrictive cardiomyopathy.

Since 2016 every child listed for HTX is carefully evaluated for an underlying disease in our institution (Figure 1). Aside from thorough clinical examination, echocardiography and cardiac MRI, this includes a myocardial biopsy in all patients with DCM to exclude or confirm an infectious origin. A definite diagnosis of myocarditis is accepted when the Dallas criteria are fulfilled [22] and a specific pathogen is identified by biopsy. If no virus is identified, but the Dallas criteria are positive, the CM is classified as potentially caused by myocarditis. A cardiac biopsy is optional for subjects with RCM, HCM or NCM. In all patients with clinical and/or paraclinical findings suggesting a systemic disorder, a metabolic work-up including at least determination of lactate, ammonia, amino acids in plasma, acylcarnitine profile, organic acids in urine, homocysteine in plasma, transferrin electrophoresis, and screening for increased oligosaccharide and glykosaminoglykan excretion in urine is performed. 

In case a biopsy does not confirm myocarditis and no clinical/paraclincal findings suggest a specific systemic disorder, Next-Generation-Sequencing (NGS) is initiated. A commercially available gene panel is applied that includes the following genes: ABCC9, ACTC, ACTC1, 1ACTN2, ANKRD1, BAG3, CALR3, CAV3,CRYAB, CSRP3, DES, DMD, DSP, FKTN, ILK, JPH2, LAMA4, LAMP2, LDB3, LMNA, MYBPC3, MYH6, MYH7, MYL2, MYL3, MYLK2, MYOZ2, MYPN, NEBL, NEXN, PLN, PRDM16, PRKAG2, RAF1, RBM20, SCN5A, SGCD, TAZ, TCAP, TMNI3, TMNT2, TNNC1, TPM1, TTN and VCL. For this study the guidelines from the American College of Medical Genetics and Genomics (ACMG) were used for the interpretation of sequence variants. Only variants that where pathogentic or likely pathogenic are presented. Variants of uncertain significance are controversely discussed [23] and are excluded in the present study.

If these investigations reveal no explanation for the patient’s CM, all relevant data are discussed in an interdisciplinary team meeting with specialists from the departments of Neuropathology, Child Neurology and Genetics. If appropriate, patients are re-examined by one of these specialists and/or additional examinations are initiated to further delineate the exact phenotype. Thereafter, a targeted genetic testing or a whole-exome-sequencing (WES) is carried out [19].

This study complies with the Declaration of Helsinki and was approved by the ethical committee of the University of Giessen. Informed consent has been obtained from the subjects (or their legally authorized representative). All data are presented as mean with standard deviation. Categorical data are expressed as counts and proportions. Data were evaluated separately for the time periods 1988–1999, 2000–2009, and 2010–2019. Kaplan-Maier survival curves were calculated for each time period and analyzed for significant differences. The rate of deceased patients was compared between subjects with systemic and cardiac-specific diseases by the fisher exact test. A *p*-value < 0.05 was considered statistically significant. Statistics were performed with program R (R Core Team 2019; R Version 3.6.1, Vienna, Austria).

## 3. Results 

52 out of 98 subjects (53%) were male. Age at HTX was <1 year in 21 patients (21.4%), 1–6 years in 38 (38.7%), 7–12 years in 13 (13.2%), and >12 years in 26 (26.5%). 70 (71.4%) subjects suffered from DCM, 10 (10.4%) from RCM, 12 (12.2%) from NCM, 5 (5%) from HCM, and one from ARVC (1%). The number of transplants due to CM increased substantially during the different time intervals, and accounted for 22% of all transplantations from 1988 to 1999, for 47.8% from 2000 to 2009, and for 53.2% from 2010 to 2019 (Table 1).

**Figure 2:**
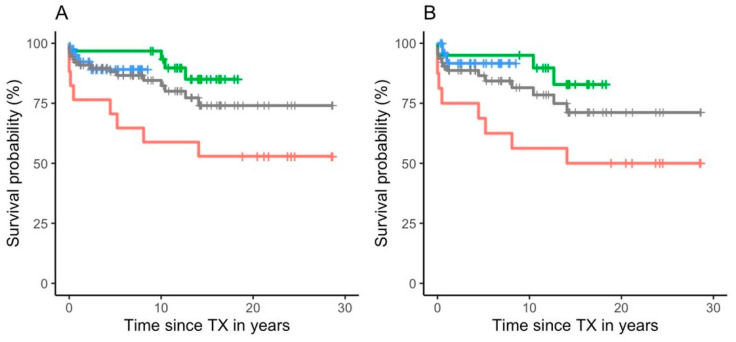
Kaplan-Mayer plots of all 98 patients with cardiomyopathy undergoing heart transplantation for three different time periods (1988–1999 = red line, 2000–2009 = green line, and 2010–2019 = blue line) (**A**), and for 70 patients with dilated cardiomyopathy alone (**B**). The gray lines correspond to the overall survival rates of all three time periods together.

The overall HTX survival rates for patients with CM were 93% after 1 year, 84% after 10, and 75% after 30 years, respectively. The survival rates for subjects undergoing HTX between 1988 and 1999 were 76% after 1, 71% after 5, 59% after 10, and 53% after 30 years. The survival numbers for individuals transplanted during the period 2000–2009 were 97% after 1 year, 97% after 10 years, and 86% after 15 years. For patients receiving a new heart in the time interval from 2010–2019 1-year survival was 96%, while 87% were still alive after 5 years (Figure 2A). The overall HTX survival rates for patients with DCM, representing by far the largest subgroup, were 91% after 1 year, 83% after 10, and 73% after 30 years. The survival rate from 1988–1999 was 75% after 1, 69% after 5 years, 56% after 10 years, and 50% after 30 years. During the period 2000–2009 95% survived 1 year, 95% 10 years, and 84% 15 years. In the time interval from 2010-2019 1-year survival rate was 97%, while 93% were still alive after 5 years (Figure 2B). Gender had no significant influence on survival.

Overall survival rates were significantly higher in the time periods 2000–2009 and 2010–2019 than in the interval 1988–1999 (*p* < 0.01), whereas no statistically relevant difference was found between the time range 2000–2009 and 2010–2019. In total, 17 out of 98 patients died (17.3%). Cause of death was related to early complications associated with HTX in 5, acute rejection in 4, and posttransplant lymphoproliferative disease in 4 patients. Cardiac allograft vasculopathy, malignant cardiac arrhythmias, and pulmonary embolism were the cause of death in one individual each. Reason for death remained unclear in one subject.

The methods applied to clarify the aetiology of CM changed over time. Virtually no testing was performed from 1988–1994, and consecutively almost all cases were classified as unknown. Myocardial biopsies were conducted since 1995, while metabolic and genetic testing were carried out at an increasing rate from 2010 on, and are routinely implemented since 2016. Myocarditis was diagnosed in 15 subjects (15.3%), potential myocarditis was suspected in 4 patients, cardiospecific defects were identified in 7 subjects (7.1%), and systemic disorders were unravelled in 15 (15.3%). Among the latter, 6 subjects (6.1%) were affected by a mitochondrial disorder. In addition, cardiotoxicity was the cause of CM in 3 patients receiving chemotherapies due to oncological diseases. (3.1%) (Supplementary Table S1A–C). Collectively, 26 patients (26.5%) were affected by a cardiac-specific disorder and 15 (15.3%) by a systemic disease including oncological diseases. Overall, the aetiology of CM could be clarified in 37 subjects (37.7%) and in 41 (41.8%) if potential myocarditis is counted as well. (Table 1; Supplementary Table S1A–C). By contrast, the sophisticated diagnostic protocol applied since 2016 yielded a detection rate of 66.6% (12 out of 18 subjects).

One out of 26 patients with a cardiac-specific disease was mentally retarded, while eight out of 12 individuals with systemic diseases had distinct neuro-cognitive deficits (*p* = 0.001) (patients with an oncological history were excluded). No subject out of 26 with cardiac specific diseases died, whereas four out of 12 with systemic diseases died (*p* < 0.05).

## 4. Discussion 

In this study, we assessed the long-term survival after HTX in children with CM and analyzed whether the cause of CM influenced mortality and morbidity. The major findings of this study were that almost three quarters of patients were still alive 30-years after heart transplant, and that neurocognitive deficits were more frequent and mortality was higher in individuals with systemic disorders compared to subjects with cardiac-specific diseases.

The Kaplan-Meier 30-year survival rate of patients with CM transplanted in our center was 74%. This number is substantially higher than the 15-year (50–75%) [10,11,12,13] and 25-year (37%) [14] survival rates reported by the ISHLT. This discrepancy can be explained by skill and competence acquired during many years of experience with HTX in a single institution, whereas pooled data are negatively influenced by inclusion of less experienced low volume centres. This interpretation is supported by the significantly higher survival probability of children transplanted within the last two decades (*p* < 0.01), presumably reflecting a learning curve due to better intensive care management and improved immunosuppressive therapy [24,25].

While the incidence of DCM is only slightly higher than that of HCM [2] the amount of children with DCM undergoing HTX in our cohort was 2.5 (70 vs 28) times higher, demonstrating the greater risk of subjects with DCM to develop terminal heart failure early in life. The survival rate of subjects with DCM alone was not distinctly different from that of all individuals with CM.

In adults, irreversible pulmonary disease or renal disorder, coexisting neoplasm, insulin-dependent diabetes mellitus with end-organ damage, active peptic ulcer disease or diverticulosis, and hepatic or systemic disease have been considered contraindications to HTX [26,27,28,29]. The ISHLT, performing a multivariable analysis to assess categorical factors with an increased risk of one year mortality for patients with CM, found that artificial ventilation or dialysis at the time of transplant, other diagnosis than DCM, and small number of patients transplanted in a center were negative predictors [30]. In divergence from adults, information about co-morbidities or systemic diseases in patients with CM younger than age 18 years is very limited, presumably reflecting that children are not routinely evaluated for factors influencing success of HTX. 

We compared the outcome of patients with cardiac-specific and systemic diseases. This differentiation seems meaningful since morbidity and mortality in this study were higher in the latter group. Identifying children with an increased mortality risk is important since it allows anticipating potential complications, and permits to avoid them at all or to treat them early (e.g., neutropenia in Barth-syndrome). Moreover, tagging of an underlying progressive disease could help in making the decision whether a child should be listed for HTX or not [31]. Notably, diagnosing a systemic and/or potentially progressive disorder does not mean principle exclusion of HTX. This is exemplified by subjects with Barth-syndrome and slowly progressive types of limb girdle muscular dystrophy in our cohort; disorders in which successful treatment of early and severe heart failure by HTX has been shown to prolong survival for many years [32]. 

One-fifth of our patients were transplanted during the first year of life, reflecting that they were affected by a rapidly progressive form of CM. Diagnosing a systemic disorder in very young subjects is challenging since other organs may become affected only later, and because symptoms characteristic of an underlying disease such as muscular hypotonia or lactic acidosis are unspecific and can overlap with features of heart failure. Moreover, the differential diagnosis of systemic and potentially progressive disorders also causing CM is very broad [33]. 

Myocarditis was the most frequent cause leading to terminal heart failure in the present investigation. In divergence to some other studies, which identified this cause as a risk factor for increased post-transplant mortality [34,35], we found no indication for higher mortality in this group. This difference can be explained by the fact that in this study a definite diagnosis of myocarditis was only accepted when the Dallas criteria were fulfilled, and a specific pathogenic agent was detected in myocardial tissue. 

Mitochondrial diseases were the leading cause of systemic CM in our cohort. About 1300 nuclear and 37 mitochondrial genes are involved in the maintenance and regulation of mitochondrial function, and currently, mutations in more than 250 genes have been shown to result in disease [36]. While in some patients, a combination of symptoms forms a characteristic syndrome (e.g., cyclic or intermittent neutropenia, muscle weakness, growth delay, 3-methylglutaconic aciduria, and CM in Barth syndrome); single organ involvement, unspecific features of multi-organ affection, and marked genetic variability impede the diagnosis in many others [33]. Therefore, it seems possible that mitochondrial dysfunction is a still underdiagnosed cause of CM related terminal heart failure.

Interestingly, two unrelated patients from our cohort were diagnosed with mutations in *GTPBP3*, encoding the mitochondrial GTP-binding protein 3, by Whole-Exome-Sequencing (WES) after HTX. Currently, the two patients’ neurocognitive development is normal and their clinical status is stable since 4 and 5 years, respectively, despite lactic acidosis, non-progressive muscle weakness, and exercise intolerance. Until now, only one study reporting 11 subjects with mutations in this specific gene is on record. In divergence from what we see in our two patients, this study suggested a rather severe course of disease with early and prominent CNS-involvement in most patients [37]. These two cases highlight the ethical problems that can arise from diagnosing a rare mitochondrial disorder underlying CM; since it would have been a difficult decision, whether to list these patients for HTX or not, if we would have had this information in advance [31]. 

Over time our diagnostic program in subjects with CM undergoing HTX has been adjusted, currently allowing unravelling the etiology of CM in about two thirds of patients. Especially NGS techniques have substantially facilitated the detection of cardiac-specific and systemic gene defects in children with CM [19]. While commercially available gene panels were well-suited to identify the genetic defect in patients with sarcomeric protein disorders, WES was the method of choice to disclose the exact cause in individuals with multisystemic diseases. A diagnostic algorithm for subjects with CM considered to be listed for HTX is proposed in Figure 2, and may lead to a higher percentage of definite underlying diagnosis as described before [38]. 

Identifying the mutation has an impact on the family as all 1st degree family members were advised for genetic evaluation. In the case a pathogenic or likely pathogenic mutation was ruled out, no further cardiologic follow up was initiated. In the case of mutations with uncertain significance, a regular follow up was planned.

Our study has several shortcomings, limiting the conclusions that can be drawn from the results. Most importantly, this was a single centre investigation including a rather small number of patients. In addition, the diagnostic work-up was not the same for all subjects. Therefore, it is well possible that the amount of disease groups and single genetic defects underlying CM as well as the number of patients with definite diagnosis will be different when a larger population is followed prospectively in a multicentre study with a uniform study protocol applying the newest diagnostic tools.

## 5. Conclusions

In conclusion, we found that the long-term survival rate of children with CM and HTX is high, but that survival probability is lower and neurocognitive deficits are more frequent in individuals with systemic disorders. A sophisticated diagnostic workup allows unraveling the basic defect in the majority of patients with CM undergoing HTX, and thus can help to identify subjects with an increased risk for organ loss. A meticulous diagnostic work-up before listing a child with CM for transplantation seems meaningful to improve risk stratification and to optimize personalized medical care.

## Figures and Tables

**Table 1:** Aetiology of CM in children necessitating HTX.

		1988–1999	2000–2009	2010–2019
All HTX patients		77	67	92
Deceased HTX patients		27	11	14
CM HTX patients	98	17	32	49
Deceased CM HTX patients	16 (16.3%)	8 (47%)	3 (9.3%)	5 (10.2%)
**DCM HTX**	70	16	19	35
Cardiac-specific				
definite myocarditis		3	4	7
potential myocarditis		1	1	2
other cardiac-specific disorder		-	1	5
systemic disorder		-	2	6
Others		-	1	2
Unknown		12	10	13
**HCM HTX**	5	0	1	4
systemic disorder		-	1	2
Unknown		-	-	2
**RCM HTX**	10	-	6	4
systemic disorder		-	-	1
Others		-	-	1
Unknown		-	6	2
**NCM HTX**	12	1	6	5
Definite myocarditis		-	-	1
Unknown		1	6	4
**ARVC HTX**	1	0	0	1
Cardiac-specific disorder		-	-	1

CM = cardiomyopathy, DCM = dilated cardiomyopathy, HCM = hypertrophic cardiomyopathy, RCM= restrictive cardiomyopathy, NCM= Non compaction cardiomyopathy, ARVC = Arrhythmogenic right ventricular cardiomyopathy. HTX = Heart transplantation.

## Supplementary Materials

The following are available online at https://www.mdpi.com/2075-4426/10/4/251/s1, Table S1A: Synopsis of patients with CM-related heart transplantation and systemic disease, Table S1B: Underlying diseases leading ta heart transplantation: Cardiac-specific diseases, Table S1C: Underlying diseases leading to heart transplantation: Myocarditis.
