# Supplementary material for: Aetiology and 30-Year Long-Term Outcome of Children with Cardiomyopathy Necessitating Heart Transplantation
Source: J Pers Med. 2020 Nov 27;10(4):251. doi: 10.3390/jpm10040251 (PMC7712803; doi:10.3390/jpm10040251)
Supplement: Supplementary file 1 [file jpm-10-00251-s001.pdf]

Supplementary Table 1A: Synopsis of patients with CM-related heart transplantation and systemic disease

Patient	Year of birth	Type of CM	Age at transplantation (d)	Death after HTX (d)	Underlying disease	Diagnostic tool*	Gene	Mutation
**1**	1983	DCM	9475	9488	Peters-Plus-syndrome	Clinical examination		
**2**	1989	DCM	4782	8587	Marfan-syndrome	Clinical examination		
**3**	1990	RCM	7592	-	Anthracycline chemotherapy	Patient history		
**4**	1991	HCM	5218	8878	Charcot-Marie-Tooth-Disease	Clinical examination		
**5**	1992	DCM	5130	-	Adriamycin chemotherapy	Patient history		
**6**	1998	DCM	6524	-	limb-girdle muscular dystrophy	Whole exome sequencing	*FKRP* *FKRP*	c.826C>A, c.1210C>T

**7**	2004	DCM	3885	-	Mitochondrial disorder	Whole exome sequencing	*GTPBP3*<br>*GTPBP3*	c.709G>T<br>c.583C>T
**8**	2007	DCM	4399	-	Daunorubicin chemotherapy	Patient history		
**9**	2008	RCM	3079	-	BAG-3-Mutation	Whole exome sequencing	*BAG 3*	c.626C>T
**10**	2010	HOCM	250	-	Noonan syndrome with multiple lentigenes	Gene panel testing	*PTPN11*	c.31528C>G
**11**	2013	HCM	895	-	Mitochondrial disorder	Whole exome sequencing	*GTPBP3*	c.506C>T<br>c1178T>C
**12**	2013	DCM	1865	-	Mitochondrial disorder	Muscle Biopsy		
**13**	2013	DCM	329	-	Mitochondrial disorder	Gene panel testing	*TAZ*	c.222 C>G
**14**	2016	DCM	109	311	Mitochondrial disorder	Muscle Biopsy		

**15**	2017	DCM	182		Mitochondrial disorder	Gene panel testing	*TAZ*	c.280 C>G

* = diagnostic tool finally solving the case

Supplementary Table 1B: Underlying diseases leading ta heart transplantation: Cardiac-specific diseases

**Patient**	**Year of Birth**	**Type of CM**	**Age at transplantation in days**	**Death after HTX**	**Underlying disease**	**Diagnostic tool***	**Gene**	**Mutation**
**1**	1996	DCM	5598	-	MYH7 Mutation	Gene panel testing	*MYH7*	c.5401G>A
**2**	2004	DCM	3827	-	MYBPC3 Mutation	Gene panel testing	*MYBPC3*	c.565G>A<br>c.706A>G
**3**	2004	ARVC	5197	-	PKP2 Mutation	Gene panel testing	*PKP-2*	c.2327C>T
**4**	2004	DCM	793	-	Troponin T2 Mutation	Gene panel testing	*TNNT2*	c.286 G>A
**5**	2006	DCM	1463	-	Long QT-syndrome	Clinical examination		
**6**	2014	NCM	1384	-	TPM1 Mutation	Gene panel	*TPM 1*	c.272G>T

						testing		
**7**	2018	DCM	331	-	TPM1 Mutation	Gene panel testing	*TPM1*	c.688G>A

* = diagnostic tool finally solving the case

Supplementary Table 1C: Underlying diseases leading to heart transplantation: Myocarditis.

**Patient**	**Year of birth**	**Type of CM**	**Age at transplantation (d)**	**Death after HTX**	**Diagnostic tool**	**Pathogen**
**1**	1980	DCM	4941	-	Cardiac biopsy	*Parvo B19*
**2**	1985	DCM	5410	-	Cardiac biopsy	*Parvo B19*
**3**	1993	DCM	514	-	Cardiac biopsy	*Coxsackie Typ B*
**4**	1995	DCM	3144	-	Explanted Heart	*Parvo B19*
**5**	1999	DCM	154	-	Maternal blood sampling	*Parvo B19*
**6**	2000	DCM	590	-	Cardiac biopsy	*Parvo B19*
**7**	2002	DCM	559	-	Explanted Heart	*HHV 6*
**8**	2007	NCM	1321	-	Cardiac biopsy	*Parvo B19*
**9**	2007	DCM	4280	-	Cardiac biopsy	*HHV 7*

**10**	2011	DCM	213	-	Cardiac biopsy	*HHV 6*
**11**	2012	DCM	430	-	Cardiac biopsy	*HHV 6*
**12**	2013	DCM	441	-	Cardiac biopsy	*Coxsackie type B*
**13**	2013	DCM	195	-	Cardiac biopsy	*Coxsackie type B*
**14**	2015	DCM	1336	-	Cardiac biopsy	*HHV 6*
**15**	2017	DCM	275	-	Maternal blood sampling	*Coxsackie type B*

Potential Myocarditis

**16**	1993	DCM	6087	-	Patients History	
**17**	1995	DCM	753	-	Cardiac biopsy	
**18**	2004	DCM	4706	-	Cardiac biopsy	
**19**	2008	DCM	5762	-	Cardiac biopsy	
